# Anthropogenic pressures drive population genetic structuring across a Critically Endangered lemur species range

**DOI:** 10.1038/s41598-019-52689-2

**Published:** 2019-11-07

**Authors:** Andrea L. Baden, Amanda N. Mancini, Sarah Federman, Sheila M. Holmes, Steig E. Johnson, Jason Kamilar, Edward E. Louis, Brenda J. Bradley

**Affiliations:** 1 Department of Anthropology, Hunter College of the City University of New York, 695 Park Avenue, New York, NY 10065 USA; 2 Department of Anthropology, The Graduate Center of the City University of New York, New York, NY 10016 USA; 3 The New York Consortium in Evolutionary Primatology (NYCEP), New York, USA; 4 Department of Ecology & Evolutionary Biology, Yale University, New Haven, CT 06511 USA; 5 Department of Anthropology and Archaeology, University of Calgary, Calgary, Canada; 6 Department of Anthropology, University of Massachusetts, Amherst, Massachusetts, 01003 USA; 7 Omaha’s Henry Doorly Zoo and Aquarium, 3701S 10th St, Omaha, NE68107 USA; 8 Center for the Advanced Study of Human Paleobiology, Department of Anthropology, The George Washington University, Washington, DC 20052 USA

**Keywords:** Conservation biology, Genetic variation

## Abstract

In recent decades Madagascar has experienced significant habitat loss and modification, with minimal understanding of how human land use practices have impacted the evolution of its flora and fauna. In light of ongoing and intensifying anthropogenic pressures, we seek new insight into mechanisms driving genetic variability on this island, using a Critically Endangered lemur species, the black-and-white ruffed lemur (*Varecia variegata*), as a test case. Here, we examine the relative influence of natural and anthropogenic landscape features that we predict will impose barriers to dispersal and promote genetic structuring across the species range. Using circuit theory, we model functional connectivity among 18 sampling localities using population-based genetic distance (F_ST_). We optimized resistance surfaces using genetic algorithms and assessed their performance using maximum-likelihood population-effects mixed models. The best supported resistance model was a composite surface that included two anthropogenic features, habitat cover and distance to villages, suggesting that rapid land cover modification by humans has driven change in the genetic structure of wild lemurs. Primary conservation priority should be placed on mitigating further forest loss and connecting regions identified as having low dispersal potential to prevent further loss of genetic diversity and promote the survival of other moist forest specialists.

## Introduction

Anthropogenically driven habitat modification is recognized as the single greatest threat to global biodiversity^1^, particularly in tropical ecosystems^2,3^. Habitat loss and fragmentation act to rapidly reduce suitable habitat area, quality, and structural connectivity (i.e., the degree to which habitat patches are physically linked by corridors) with potentially dramatic consequences for population viability^4–6^. The extent to which fragmentation impacts functional connectivity, however — that is, whether structurally intact corridors *actually* facilitate movement between habitat patches (see ref.^7^ for review) — depends largely on a species’ biology and behavior (e.g., locomotor patterns, ecological specializations, and/or microhabitat/matrix use) (reviewed in refs^8–10^). While some organisms may thrive in anthropogenically altered habitats and/or can easily traverse a matrix with low structural connectivity (e.g., rhesus macaques^11^; Norwegian rats^12^; white-footed mice^13^; spotted salamanders^14^; chimpanzees^15^), others may be more sensitive to natural and/or anthropogenic change and quickly become both geographically and genetically isolated (e.g., western gorillas^16^; Australian squirrel gliders^17^; white-fronted chat^18^; reviewed in ref.^9^). Fragmented populations may then face rapid declines in size, demographic isolation, and reduced genetic variation^6,19^. In the long-term, they may also face inbreeding depression, reduced evolutionary potential, and increased extinction risk^20–22^. Local extinctions of small isolated populations are relatively common^23,24^ and thus a species’ persistence depends largely on its dispersal ability, thereby maintaining functional connectivity among its remaining populations^25,26^.

Landscape genetic methods allow researchers to quantify the dispersal ability of a species by examining the relative effects of landscape composition, configuration, and matrix quality (*sensu* ref.^27^) on the spatial patterning of neutral and adaptive genetic variation among individuals and within populations^28–30^. The landscape genetics paradigm is a relatively recent improvement over more traditional population genetics methods in that it incorporates spatially explicit data to investigate the influence of landscape heterogeneity on gene flow and genetic variation within and among populations^29,31^. In so doing, landscape genetic analyses enable researchers to identify suitable areas in the matrix (i.e., corridors) that promote gene flow and maintain landscape connectivity^31–33^.

Until recently, landscape genetic studies have typically used expert opinion to parameterize resistance values for the landscape features in question^34^, and less frequently empirical movement studies (e.g., ref.^35^) or spatial predictions of ecological processes^36^. Although acceptable approaches, they suffer in that they test a limited resistance space (typically determined *a priori*), and resistances are not objectively assessed prior to model building. Expert opinion is often insufficient to accurately predict movement (e.g., refs^37,38^). In fact, even when ecological processes are known and modelled, there is no guarantee that these processes will be meaningful to animal movement and gene flow^36^. Recently, however, Peterman^39^ developed a more objective method to assign resistance values to both continuous and categorical landscape layers using genetic algorithms. This technique enables the parameterization of landscape surfaces with no *a priori* information about the direction or magnitude of the effect a landscape feature has on a species’ movement. The use of genetic algorithms for parameterizing resistance surfaces allows for a wider search of the parameter space for possible resistance values. New analytical tools (e.g. *ResistanceGA*^39^) allow for novel implementation of this optimization and assignment method, along with a selection criterion to discern between single and composite surfaces using linear mixed effects models fit with the maximum-likelihood population effects (MPLE) parameterization^40^. Here, we incorporate these new methods to explore the impacts of landscape heterogeneity on gene flow and genetic structure in Madagascar, using Critically Endangered black-and-white ruffed lemurs (*Varecia variegata*), which range along the eastern rainforest corridor, as an illustrative test case.

Madagascar is among the most diverse and the most threatened biodiversity hotspots in the world^41^. As such, it is often identified as a global conservation priority^41–43^. Since the 1950s, approximately half of Madagascar’s remaining moist forest has been lost to anthropogenic habitat modification; its remaining forest habitats continue to face rapid conversion and associated fragmentation which threaten the country’s unique biota^44,45^. Ruffed lemurs are relatively large-bodied^46^ (3.5–4.5 kg), arboreal, obligate frugivores that are exclusive to Madagascar’s eastern rainforest corridor^47^, though they are thought to have historically ranged as far south and west as Toliara, roughly 415 km southwest of their southernmost distribution today^48^. Although known to persist in degraded habitats^49^ and forest fragments^50–52^, the species exhibits a preference for low- to mid-altitude primary rainforest^47^. Ruffed lemurs live in large social groups^53^, maintain large home ranges^54,55^ (e.g., 87.8–90.5 ha), and rely on a spatiotemporally patchy diet^56^. The species dominates the upper canopy and rarely comes to the ground^57^; thus, dispersing individuals are unlikely to cross an open landscape matrix. Moreover, ruffed lemurs are highly selective feeders, and can be considered ecological specialists^56–58^, making them especially vulnerable to habitat modification^49,59^. In fact, they are among the first taxa to disappear in the face of habitat disturbance^60^. Consequently, remaining populations of this species are fragmented into several geographically distinct localities with seemingly limited potential for reproductive contact^61^.

Recent studies have found low levels of genetic diversity among the remaining ruffed lemur populations and high levels of genetic differentiation^52,61^. These patterns differ geographically, in that northern sites are characterized by greater genetic diversity and gene flow than southern localities^61^. It is possible that these differences are related to landscape connectivity; however, the extent to which different landscape features impact dispersal and gene flow in this species remains unexplored.

Here we investigate the relationships between genetic structure and landscape features to identify factors that impede or facilitate ruffed lemur dispersal (i.e., functional connectivity) on a range-wide scale. We test the null hypothesis of isolation-by-distance and then consider the effects of five potentially related landscape features that we predict will impose barriers to dispersal and promote genetic structuring among sampling localities: (1) rivers, (2) topography, (3) roads, (4) habitat cover, and (5) proximity to human activity. Natural barriers such as rivers have been linked to the genetic structuring of several vertebrate taxa (e.g., red grouse^62^; mountain lions^63^; bonobos^64^; western gorillas^16^; radiated tortoises^65^) and have been argued to play a particularly important role in the biogeography of Malagasy strepsirrhines^66–69^. We therefore expect rivers to explain a large proportion of the genetic differentiation found among sites. Additionally, because ruffed lemurs are arboreal specialists, are ecologically constrained, and prefer primary rainforest habitats^47^, we predict that anthropogenic pressure in the form of roads, reduced habitat cover, and proximity to human habitation will impose significant barriers to gene flow. Finally, although ruffed lemurs prefer undisturbed, low- to mid-altitude forest, the species has been shown to persist in degraded habitats^49–52^ and at higher elevations^70^ (e.g., Mangevo range: 800–1200 m altitude). Thus, although we expect forest degradation and elevation to have some influence on population genetic structure, these are predicted to show weaker relationships with genetic distance than other landscape features identified above. To our knowledge, ours is only the second study to use both Peterman’s^39^ recent method of resistance surface optimization with genetic algorithms and maximum-likelihood population-effects (MLPE) mixed models^40^ for model selection of a wild, endemic vertebrate across its entire known geographic extent, and the first to do so in Madagascar. Using newly developed landscape genetics methods that more objectively identify resistance surfaces and resistance surface complexity should yield more robust results compared to prior approaches.

## Materials and Methods

### Genetic sampling and genotyping

**Figure 1:**
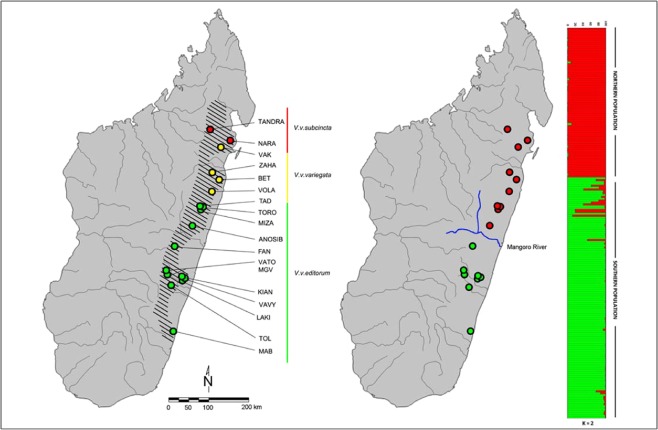
Map illustrating locations of the n = 18 sampling localities included in this study (left). Colored nodes correspond to current subspecies status, as indicated to the right of the map. We provide results from an earlier structure plot (right) modified from Baden *et al*. (2014), which identify the Mangoro River as a likely driver of *V*. *variegata* population genetic structure into northern (red) and southern (green) genetic clusters.

We used the original dataset from Baden *et al*.^61^, excluding nine individuals from the introduced island population from Nosy Mangabe. These data included a panel of 10 microsatellite loci for 200 adult black-and-white ruffed lemurs (*Varecia variegata*) (99 males, 101 females) sampled from 18 localities spanning the species’ known range (Fig. 1). Provenience of each sample was reported to the level of sampling locality (i.e., all individuals from a locality have the same geographic coordinates). We assigned Cartesian coordinates to each population based on the centroid of the sampling localities. We estimated pairwise genetic dissimilarity among the 18 populations in our sample by calculating Wright’s Fixation Index (F_ST_) using the program FSTAT2.9.3.2^71^. Given that continuous sampling was not possible at such a broad range, population-based metrics were chosen over individual-based statistics^72^.

### Connectivity and circuit theory

We used isolation-by-resistance *sensu* McRae^73^ to model functional connectivity amongst sites. The isolation-by-resistance (IBR) model predicts a positive relationship between genetic distance and the resistance distance, a graph theoretic distance metric based in circuit theory^73^. Circuit theory is a theoretical framework that treats landscapes as conductive surfaces, using electrons flowing across an analogous circuit to model individuals or genes moving across the landscape^74^. Circuit theory simultaneously considers all possible pathways between two sampling localities when assessing landscape influence on gene flow, thus allowing for a more thorough investigation of these processes than least cost path (LCP) analysis^74,75^ (but see ref.^76^). We calculated circuit-based connectivity using Circuitscape 4.0^77^. To do so, we developed five resistance surfaces (see details below) and assessed these in relation to the population-based pairwise genetic distance, F_ST_.

### Remote sensing and landscape feature selection

**Figure 2:**
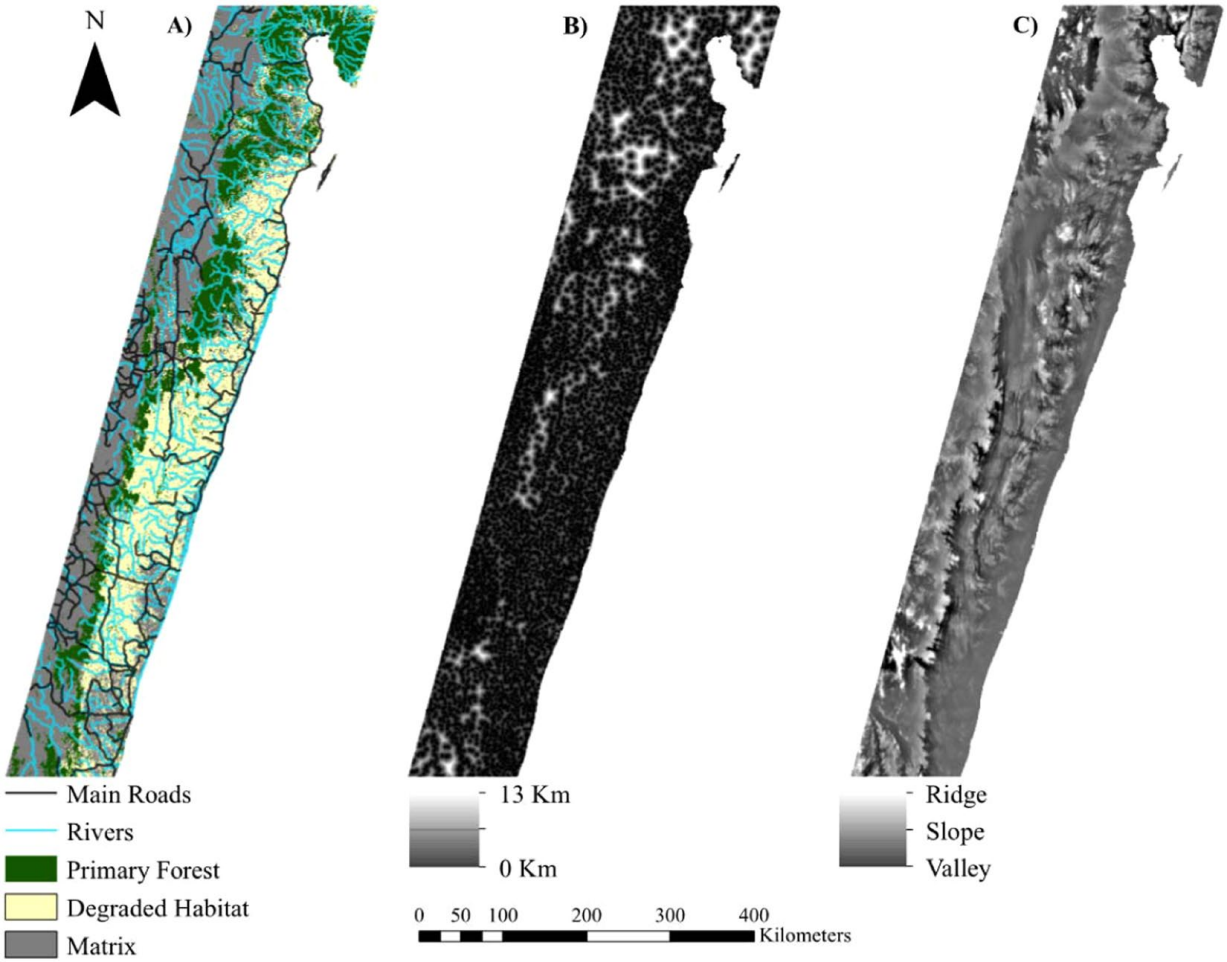
Maps of eastern Madagascar showing the landscape surfaces for (**A**) habitat, rivers, and roads; (**B**) distance to nearest village; and (**C**) topographic position index (TPI). Habitat type was derived by reclassifying a vegetation raster from The CEPF Madagascar Vegetation Mapping Project (http://www.vegmad.org/), while river and road data were downloaded from DIVA-GIS (http://www.diva-gis.org/), clipped to include only major features (i.e., using only roads that had been designated as ‘roads’ rather than ‘trails’), and then rasterized. Distance to nearest village was generated by selecting point locations with a designation of “Populated Places” from a gazetteer of foreign geographic feature names (data was downloaded from DIVA-GIS at http://www.diva-gis.org/gdata; source: GEOnet Names Server at the U.S. National Geospatial-Intelligence Agency), and calculating straightline distances from each site to the nearest village locale. Topographic position index was generated from a 30 arcsecond resolution digital elevation model (DEM; downloaded from DIVA-GIS at http://www.diva-gis.org/) using the Topography Tools toolkits in ArcGIS v10.3.1.

We selected five landscape features hypothesized to influence ruffed lemur movement and gene flow: (1) rivers, (2) topography, (3) roads, (4) habitat type, and (5) proximity to human habitation (Fig. 2). Categorical surfaces analyzed included habitat type (primary forest, degraded habitat, and matrix), rivers, and roads, while continuous surfaces included topographic position index (TPI), and distance to nearest village (in meters). Habitat type was derived by reclassifying a vegetation raster from The CEPF Madagascar Vegetation Mapping Project (http://www.vegmad.org/) to three primary habitat types such that primary forest represented a combination of humid and littoral forests, degraded habitat was derived from the degraded forest classification, and matrix was derived from all other categories. River and road data were downloaded from DIVA-GIS (http://www.diva-gis.org/), clipped to include only major features (i.e., using only roads that had been designated as ‘roads’ rather than ‘trails’), and then rasterized. Topographic position index, a measure that compares the elevation of each cell in the raster to the adjacent landscape and calculates a quantitative value that is indicative of the cell’s relative position (i.e., slope, valley, plain, or ridge), was generated from a 30 arcsecond resolution digital elevation model (DEM; downloaded from DIVA-GIS at http://www.diva-gis.org/) using the Topography Tools toolbox in ArcGIS v10.3.1^78^. Village locations were obtained by selecting point locations with a designation of “Populated Places” from a gazetteer of foreign geographic feature names (data was downloaded from DIVA-GIS at http://www.diva-gis.org/gdata; source: GEOnet Names Server at the U.S. National Geospatial-Intelligence Agency). Village proximity was derived by calculating the distance of each cell in a 1 km resolution raster to the nearest village center. All surfaces were converted to a uniform geographic coordinate system (Universal Transverse Mercator; UTM), resampled at a resolution of 1 km, and clipped to the study extent for analysis. Previous work has shown that changes in spatial resolution do not significantly alter the results of landscape genetic analyses^74,79^. Therefore, this resolution was chosen as a trade-off between retaining detail across the landscape and minimizing processing time for analyses.

Distance is implicitly incorporated into the resistance distances calculated by Circuitscape, and thus straightline Euclidean distance between sites (i.e., geographic distance) was not included as an additional factor in our models. We did, however, conduct a linear regression between pairwise Euclidean and genetic (F_ST_) distances to identify the proportion of our data influenced by geographic distance alone.

### Landscape resistance parameterization

Resistances between sampling localities were calculated in Circuitscape 4.0^77^ based on average pairwise resistances using an eight-neighbor connectivity scheme and optimized using the R package *ResistanceGA*^39^. *ResistanceGA* utilizes genetic algorithms to adaptively search a broad parameter space to determine the optimal resistance values that best describe pairwise genetic differentiation (in our study, F_ST_). Inspired by principles of biological evolution, genetic algorithms create a population of individuals with traits (parameters to be optimized) encoded on “chromosomes”. The fittest individuals from each generation (i.e., those with genotypes or parameter combinations that solve the fitness function) survive to reproduce^80^. Parameter space is explored via ‘mutations,’ whereby new parameter values are generated, as well as via ‘crossover’, whereby genetic information is exchanged. The population continues to evolve – that is, parameterization of the resistance surfaces run – until a sufficient number of generations have passed without an improvement in fitness^81^ (see ref.^39^ for details). This approach makes no *a priori* assumptions about the direction or magnitude of the resistance between landscape and genetic distances, allowing for a more thorough investigation of the relationship between landscape features and gene flow than more widely used methods (i.e., expert based value assignments^34,82^).

Continuous surfaces were optimized using Monomolecular and Ricker transformations, while categorical surfaces were optimized by holding one feature constant at a value of 1 and then adjusting resistance values for all other features between the values of 0 and 3500 following Peterman *et al*.^36^. Genetic algorithm settings can be found in Supplementary Materials.

### Resistance optimization and model selection

We evaluated the resistance optimization process for each surface (i.e., landscape feature) using AICc (Akaike’s Information Criterion corrected for small/finite population size^83^), which was determined from linear mixed effects models with MLPE parameterization^84^ and evaluated by maximum likelihood in lme4^85^. To account for our uneven sampling design, we conducted bootstrap resampling using 75% of the data (13 sampling locations) to control for bias following Ruiz-Lopez *et al*.^86^; these data were randomly selected without replacement and then optimized surfaces were fit to the selected data. Following 10,000 iterations, average rank and average model weight ($\bar{\omega }$) were determined for each resistance surface, along with the frequency with which a surface was ranked as the top model ($\hat{{\pi }}$) in order to address uncertainty in the top model; Burnham and Anderson^87^ identify $\hat{{\pi }}$ as the bootstrap equivalent of $\bar{\omega }$. Following the identification of the top surfaces in isolation, we ran a Spearman’s rank correlation between the surfaces to assess degree of correlation. We created and optimized composite surfaces by combining the top models; surfaces that had both a greater selection frequency ($\hat{{\pi }}$) than distance alone and were selected more than one percent of the time ($\hat{{\pi }}$ > 0.01) were used to generate composite surfaces. All single and composite surface optimization processes were conducted at least twice as per recommendations by Peterman^39^ to ensure convergence on  the top model(s). Following optimization, we again conducted a bootstrap model selection using 10,000 iterations and average rank, $\bar{\omega }$, and $\hat{{\pi }}$ were calculated in order to assess composite models in relation to their component surfaces. Finally, current flow across the landscape was visualized in Circuitscape using the best supported resistance surface.

Landscape surfaces generated during the current study are available in the Zenodo repository, as is all code used to parameterize, optimize, and asses resistance layers [http://doi.org/10.5281/zenodo.3519173].

## Results

### Landscape genetic analysis

We found a significant signature of isolation-by-Euclidean distance, which explained 28.2% (Pearson’s *r = *0.531) of the observed population genetic structure (Fig. S1). Despite evidence of IBD, two surfaces – habitat cover and distance to nearest village – were much more strongly associated with genetic differentiation than geographic distance, with the former two variables having average model weights more than 28 times higher than the latter (Tables 1 and S1). Resistance to forest cover was lowest in primary forest habitat, slightly higher in matrix, and highest in degraded landscape (Fig. S2); resistance to human habitation decreased with increasing distance from the nearest village, but increased again after approximately 8 km (Fig. S2). The remaining three surfaces (TPI, roads, and rivers) explained slightly more variation than distance, but were seldom chosen as the top model (less than 0.21% of the time) and thus were not considered in the composite analysis.

**Table 1:** Results from bootstrap selection of optimized linear-mixed effects models on single surfaces.

Surface	K	Avg. rank	$\bar{{\boldsymbol{\omega }}}$	$\hat{{\boldsymbol{\pi }}}$
Distance to Nearest Village	3	1.5855	0.366392	0.4401
Landscape Cover	4	1.8005	0.499913	0.5569
Rivers	3	3.1204	0.052107	0.0021
Roads	3	4.1946	0.034615	0.0002
Topographic Position Index	3	4.3786	0.034022	0.0007
Euclidean Distance	2	5.9204	0.012951	0.0000

K = number of parameters following continuous surface transformation or number of categories in categorical surfaces; Avg. rank = average model rank following 10,000 bootstrap iterations; $\bar{\omega }$ = average model weight averaged over 10,000 bootstrap iterations, representing the probability that the model is the best of the set; $\hat{{\pi }}$ = proportion of bootstrap iterations in which model was chosen as the top model.

**Figure 3:**
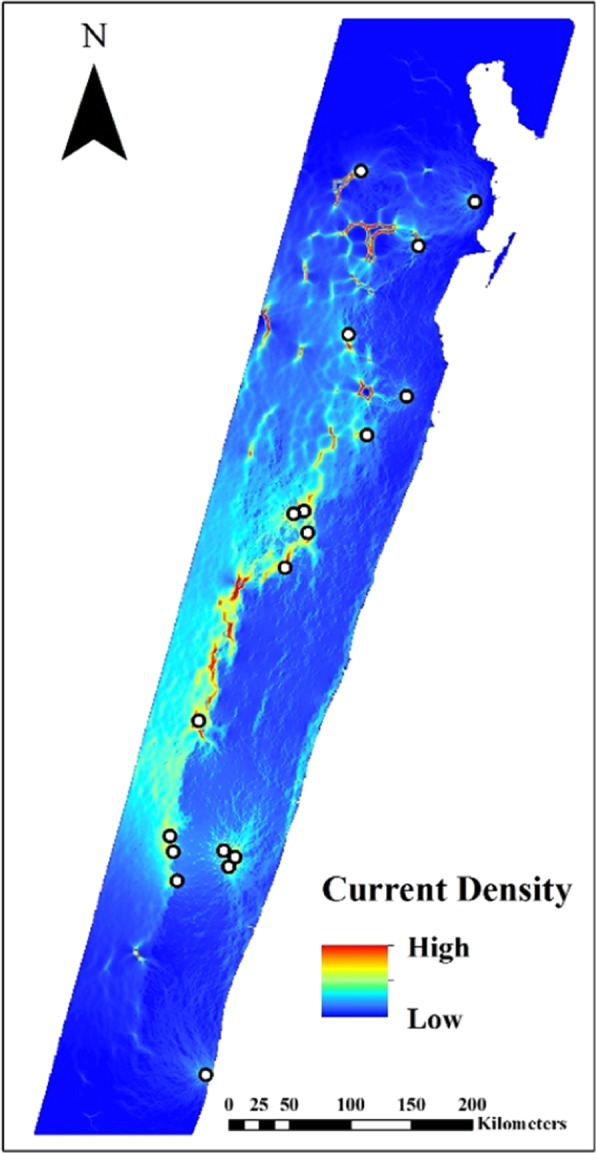
Cumulative resistance surface among sampling localities created in Circuitscape 4.0 (https://circuitscape.org). Warm colors indicate areas of high conductive value (i.e., low resistance, high dispersal ability); cool colors indicate areas of low conductive value (i.e., high resistance, low dispersal ability).

To assess the combined effects of habitat cover and distance to the nearest village, we created a composite surface that combined both habitat cover and distance to the nearest village and assessed this against the isolated surfaces, as well as straight-line Euclidean distance alone. As with its component surfaces, resistance in the composite surface decreased as distance from the nearest village increased but then increased again over large distances. Resistance in matrix was lower than, albeit almost equal to, primary forest, with degraded habitat again showing the highest resistance. The composite surface had the greatest average rank, highest average weight ($\bar{\omega }$), and was chosen as the top model 70% of the time, lending support for the composite model as the best predictor of the genetic data (Table 2). Our visualization of the composite model (Fig. 3) shows a high degree of current flow through remaining sections of *V*. *variegata*’s habitat across its range − particularly in the central region, where gene flow is likely the greatest – while also illuminating areas of near complete isolation in the south. Regions that are covered in primary forest and are distant from villages tend to be where current density is the greatest, such that the primary flow of current is located in the contiguous forest corridor stretching through the center of the existing *V*. *variegata* range.

**Table 2:** Results from bootstrap selection on optimized linear-mixed effects models on composite surface and its component single surfaces

Surface	K	Avg. rank	$\bar{{\boldsymbol{\omega }}}$	$\hat{{\boldsymbol{\pi }}}$
Composite	7	1.3028	0.524205	0.7004
Landscape Cover	4	2.3217	0.248352	0.1368
Distance to Nearest Village	3	2.4097	0.217474	0.1628
Euclidean Distance	2	3.9658	0.009968	0.0000

K = number of parameters following continuous surface transformation or number of categories in categorical surfaces; Avg. rank = average model rank following 10,000 bootstrap iterations; $\bar{\omega }$ = average model weight averaged over 10,000 bootstrap iterations, representing the probability that the model is the best of the set; $\hat{{\pi }}$ = proportion of bootstrap iterations in which model was chosen as the top model.

## Discussion

Recent studies have identified a widespread, latitudinally-structured phylogeographic pattern in several of Madagascar’s fauna, including its humid-forest mammals^88^. Several factors may be driving this pattern, including past episodes of forest contraction and expansion and biogeographic barriers (i.e., rivers or valleys). These landscape features are thought to operate on much deeper time scales than anthropogenic change in terms of their influence on an organism’s dispersal abilities, population connectivity, and genetic structure^89^. In an earlier study, we identified genetic clustering on either side of the Mangoro—the largest river in eastern Madagascar—lending support to these findings, and leading us to hypothesize that Madagascar’s rivers structure *V*. *variegata* genetic diversity^61^. We were therefore surprised to find little support for this hypothesis. Rather, our results suggest that anthropogenic factors are the primary drivers of ruffed lemur genetic structure.

There is some debate surrounding the extent to which rivers present complete barriers to vertebrate movement (e.g.,^90–95^), particularly in montane species where river headwaters are still relatively small^96^. However, rivers have been strongly implicated in the biogeography of Malagasy fauna, particularly primates^66–69^, and it is unlikely that *Varecia* are exempt from these patterns. Thus, we propose two alternative explanations for this finding. On the one hand, our results may be a product of methodological constraints, including our inability to filter our waterways landscape layer to include only the largest rivers (such as the Mangoro), as freely available data for this feature do not contain sufficient metadata to differentiate between major and minor waterways. Alternatively, we argue that natural signatures of migration and drift are being swamped by patterns caused by anthropogenic activities.

Madagascar is experiencing exceptional threats to its biodiversity^97^. In particular, deforestation has reduced forest cover by 44% between 1953 and 2014^45^, and is continuing at a pace that is projected to eliminate all of Madagascar’s eastern rainforest by 2077^70^. Of the lemurs, ruffed lemurs are particularly sensitive to anthropogenic pressure, and are among the first taxa to disappear in light of habitat disturbance^49,60^. Here, we offer further evidence of this, by presenting results indicating that anthropogenic factors alone are driving current patterns of ruffed lemur genetic structure, a finding which has important consequences for their long-term viability.

Animals are more likely to disperse through landcover that closely reflects the habitat in which they evolved^98^. Our results further support this notion, with ruffed lemur dispersal capacity being at its highest in intact primary forest, followed by matrix and degraded forest. That matrix (e.g., agriculture, grassland) proved to be a more suitable conduit to gene flow was surprising, but this finding is likely driven by the orientation of matrix to our ruffed lemur sampling localities. In our study, very few sampling locations were directly separated by matrix. Rather, with the exception of a few sites (e.g., Kianjavato, Manombo), samples were collected from protected, primary rainforest sites that were separated from the surrounding matrix by intervening degraded forest habitat. For this reason, black-and-white ruffed lemurs (or at least those included in this study) rarely encounter matrix, and it therefore did not impose a significant resistance to their movement and dispersal.

Still, it was surprising that matrix proved more permeable than degraded habitat. It is possible that this is an artefect of the classification system used, and that degraded habitat in this classification scheme represents what might be perceived as ‘matrix’ to ruffed lemurs (i.e., low quality, small-crowned fruiting trees and shrubs), effectively serving as an additional barrier to movement between patches. There is, however, indirect evidence that individuals may cross highly degraded regions at short distances, such as those that segregate rainforest fragments^52^.

The exact dispersal capacity of black-and-white ruffed lemurs is at present unknown; however, maximum mammalian dispersal distances have been shown to scale isometrically with home range size^99^. The average individual black-and-white ruffed lemur home range is ~15–20 ha^54,55^, giving an inferred migration capacity of no more than 200 km. This indicates a large dispersal capacity that would likely reduce any time lag exhibited by historic landscapes. Furthermore, all nearest neighbor sampling localities were located within this maximum dispersal distance, suggesting a high likelihood that ruffed lemurs reach at least one or more sampling locations during dispersal events. Given the orientation of the existing range for this species, however, the distances between non-neighboring sampling localities (up to 861.74 km) was often beyond the assumed functional dispersal distances of the taxon – a sampling strategy that may have decreased the signature of landscape effects on gene flow during population-level analyses^100^. At present, there are clear limitations in researcher ability to directly observe or test ruffed lemurs’ dispersal capacity. Indeed, our analysis is among the first to quantitatively relate landscape features to a measure of dispersal using genetic data. With rapid improvements in telemetry, it is now feasible to track individuals via GPS collars long-term, allowing for the possibility of capturing dispersal events and gathering spatially-explicit movement data moving forward (EEL personal observation).

As with habitat degradation, we also found increased resistance to movement with increasing proximity to human habitation. Curiously, gene flow increased again after approximately 8 km of isolation; this is likely because most villages in Madagascar are situated well within 8 km of forest, such that dispersing ruffed lemurs would rarely encounter habitat located at distances greater than 8 km from human habitation.

Unfortunately, we were unable to test the impacts of historical forest cover on the genetic structure of ruffed lemurs, as landscape maps such as these are not georeferenced and publicly available. This is an important consideration, given that historical habitat loss may yet impact future primate populations due to time lags in population responses^101^. Several factors influence the degree to which observed genetic structure reflects contemporary versus historical landscapes, including generation time, dispersal distance, and population size and demographics of the study species, as well as genetic metrics chosen for analysis^102^. Black-and-white ruffed lemurs have relatively fast generation times for primates of their body size (approximately 7–8 years^52^), suggesting that an influence from historic landscape features may no longer be detectable in contemporary genetic structure^102^. Moreover, although forest loss was already well-advanced as early as the 1600s, most significant habitat modification has occurred in the past 70 years^44,45^, suggesting that signatures of habitat modification have predominated as drivers of genetic structure in ruffed lemurs in less than ten generations. With vagile species, this generation time has shown to be sufficient for detecting newly introduced barriers to gene flow^103^.

### Conservation implications

To date, the vast majority of landscape genetic analyses have been conducted in temperate locations^31^, with a significant underrepresentation of tropical regions despite the vast majority of worldwide biodiversity being found therein^41^. Madagascar is considered both a biodiversity hotspot and a conservation priority due to its high levels of endemism in most taxonomic categories for a relatively small geographic area and extremely high loss of primary habitat^41^. Presently, over 90% of endemic lemur taxa are classified as Vulnerable and anthropogenic activities have long been implicated as a primary cause for taxonomic decline^104^. Given their sensitivity to habitat degradation, ruffed lemurs are often used as an indicator species and present an opportunity to assess the influence of anthropogenic activities across a large region of Madagascar. Our investigation suggests that rapid land cover modification by humans (e.g., refs^45,105^) has driven change in genetic structure of this indicator species, masking any signatures of natural influences.

At present, Madagascar’s natural forests cover approximately 8.9 Mha (15% of the national territory) and include 4.4 Mha (50%) of moist forests; however, estimated annual deforestation rates have progressively increased since 2005, and approximately half of Madagascar’s forest (46%) is now located at less than 100 m from the forest edge^45^. Connecting regions of low dispersal, particularly those in the southern and northern-most sampling sites, should therefore be targeted as a primary conservation priority in order to connect currently isolated ruffed lemur populations. Prior studies indicate that increased fragmentation and/or small fragment size are related to increases in genetic differentiation and lowered genetic diversity (e.g., refs^52,106,107^). Higher levels of genetic variability have been described in the northern ruffed lemur populations; however, forest loss and fragmentation have continued in this region and across the country at rates of up to 2.5% habitat lost per year since the collection of these data^105^. Additionally, northern populations experience greater intensities of hunting pressures compared to southern populations^108^, which may drive extreme losses in population size and further reduce genetic variability and increase population differentiation through greater levels of inbreeding or local extinctions. Hunting in southern localities has been recently recognized as a threat to populations as well^109^. Further investigations into the local drivers of genetic structure in this taxon are currently underway and will clarify the regional impacts of natural features and landcover modification on more immediate dispersal capacity, as well as more explicitly assess the impacts of historical landcover of observed genetic structure in this taxon.

Our study uses ruffed lemurs as a test case; however, they also represent a dispersal-, resource-, and area-limited ‘umbrella’ species (*sensu* refs^110,111^) whose habitat requirements for persistence are believed to encapsulate those of an array of associated species. As such, it is likely that the trends we see here may be echoed by other moist forest specialists, either currently or in the near future. One somewhat controversial approach might therefore be to promote ruffed lemurs as conservation ‘flagships’ (*sensu* ref.^112^) for Madagascar’s eastern rainforest corridor. Although this concept has faced some criticism (e.g., refs^113–115^), there is nevertheless evidence of greater ‘willingness-to-pay’ among private sponsorship programs for conservation focusing on charismatic species^116^. Ruffed lemurs, the largest living members of the lemurid family, can be easily recognized by their “especially long, luxuriant coat relative to other lemurs”^117^ and their suspensory feeding acrobatics^118^; they may therefore be appropriate ‘Cinderella (flagship) species’ *sensu* Smith *et al*.^119^ that can be used to attract public support for conservation efforts. Planning conservation action around this taxon, both logistically and for promotional purposes, may be one strategy toward protecting the diverse eastern rainforest species in Madagascar. These and other novel solutions will become increasingly important, as anthropogenic activities including those demonstrated herein continue to overwhelm the impact of natural processes on ecology with far-reaching, long-term and cascading consequences for the whole Earth system^120,121^ (reviewed in ref.^122^).

## Supplementary information

Supplementary Tables & Figures
