# Supplementary material for: Anthropogenic pressures drive population genetic structuring across a Critically Endangered lemur species range
Source: Sci Rep. 2019 Nov 7;9:16276. doi: 10.1038/s41598-019-52689-2 (PMC6838192; doi:10.1038/s41598-019-52689-2)
Supplement: Supplementary file 1 — Supplementary Tables & Figures [file 41598_2019_52689_MOESM1_ESM.pdf]

Supplementary Tables & Figures for:

Anthropogenic pressures drive population genetic structuring across a Critically Endangered lemur species range.

Andrea L. Baden[1,2,3*], Amanda N. Mancini[2,3], Sarah Federman[4], Sheila M. Holmes[5], Steig E. Johnson[5], Jason Kamilar[6], Edward E. Louis, Jr.[7], Brenda J. Bradley[8]

Figure S1. Linear regression between pairwise Euclidean and genetic (F_{ST}) distances; Isolation-by-distance relationship is significant (mean, 95% CI: 0.000281, 0.0002089816-0.0003531079). R-value = 0.531 (multiple R^2= 0.2822)

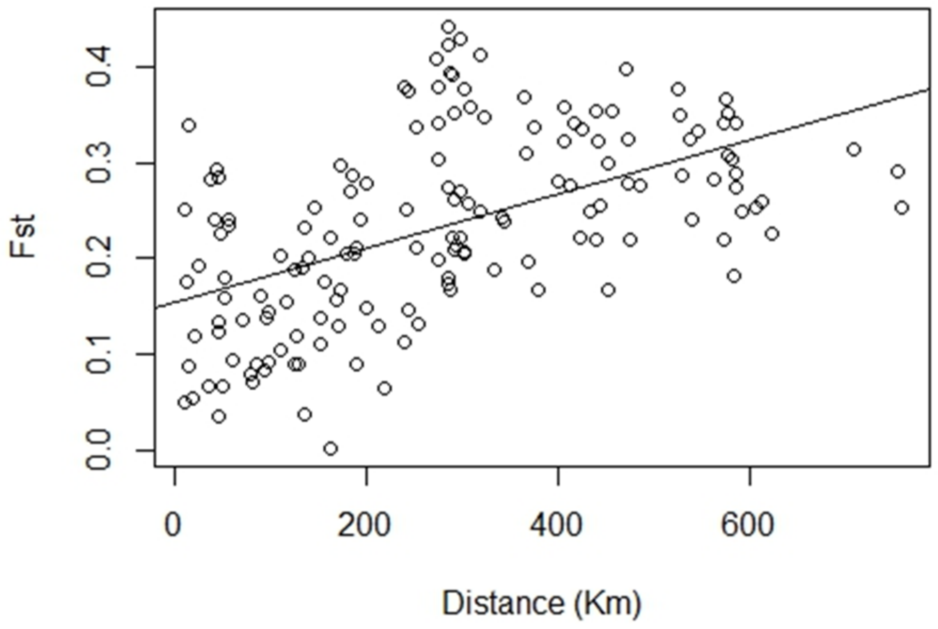

Figure S2. Plots from SS transformations; a) TPI and b) Distance to Nearest Village. The curvilinear shape to the distance to village x resistance plot (S2b) may indicate positive spatial correlation in the data, suggesting may be a threshold distance beyond which other villages are influencing there may be overlapping fields of anthropogenic effects (i.e., multiple villages) on Varecia resistance. Follow-up studies will investigate these relationships.

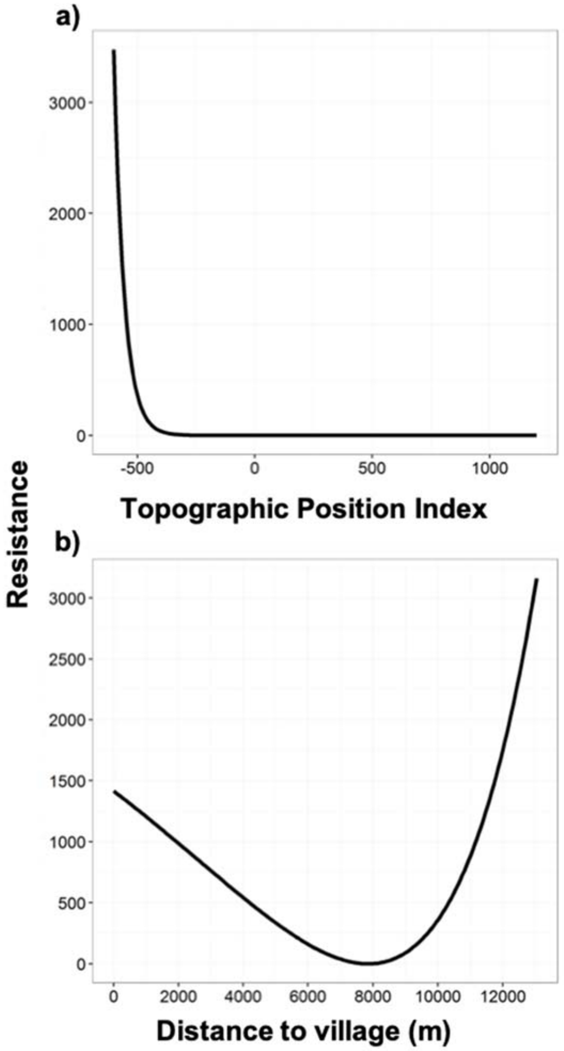

Table S1. Parameter estimates from mixed effects model fit to the optimized singular resistance surfaces. All resistance values were scaled and centered by subtracting the mean and dividing by the standard deviation of resistance values. Rows shown in bold indicate models that performed better than Euclidean distance alone and were selected as the top model more than 1% ($\hat{\pi} \geq 0.01$) during 10,000 bootstrap iterations. Values within AICc of the best model should be considered as equivalent

Resistance Surface	Parameter	β	S.E.	t-value	AICc
**Habitat Cover**	Intercept	0.234	0.023	10.207	
	**Forest cover**	**0.084**	**0.005**	**16.148**	**-446.004**
**Dist. to Nearest Village**	Intercept	0.234	0.022	10.846	
	**Distance to village**	**0.072**	**0.005**	**15.679**	**-442.234**
Rivers	Intercept	0.234	0.024	9.9485	
	Rivers	0.070	0.005	15.142	-434.290
Roads	Intercept	0.234	0.025	9.5393	
	Roads	0.073	0.005	15.182	-433.697
TPI	Intercept	0.234	0.025	9.379	
	TPI	0.070	0.005	15.235	-433.747

Table S2. Parameter estimates from mixed effects model fit to the optimized composite resistance surface and its component surfaces. All resistance values were scaled and centered by subtracting the mean and dividing by the standard deviation of resistance values. Bolded results indicate the best supported model following 10,000 bootstrap iterations (Table 3).

Resistance Surface	Parameter	β	S.E.	t-value
**Composite**	**Intercept**	**0.234288**	**0.02038**	**11.49578**
	**Forest cover x Distance to village**	**0.076023**	**0.00466**	**16.31403**
Habitat Cover	Intercept	0.234288	0.022954	10.20664
	Forest cover	0.083817	0.005191	16.14787
Distance to Nearest Village	Intercept	0.234288	0.021602	10.84553
	Distance to village	0.072129	0.0046	15.67937
